# Low-temperature two-stage probiotic fermentation enhances nutrition and safety of pig liquid feed

**DOI:** 10.1007/s00253-025-13633-9

**Published:** 2025-11-24

**Authors:** Aoran Zhang, Yuheng Cao, Yunfan Zheng, Limei Sun, Wang Yin, Jie Yu, Bing Yu, Lei Yan, Xue Yan, Yunxiang Liang, Xiaoqing Pu, Yunfang Song, Aibing Yu

**Affiliations:** 1 State Key Laboratory of Agricultural Microbiology, College of Life Science and Technology, Huazhong Agricultural University, Wuhan, 430070 Hubei China; 2 Key Laboratory of Feed and Livestock and Poultry Products Quality & Safety Control, Ministry of Agriculture and Rural Affairs, New Hope Liuhe Co., Ltd, Chengdu, 610023 Sichuan China; 3 Animal Nutrition Institute, Sichuan Agricultural University, Chengdu, 611130 Sichuan China

**Keywords:** Fermentation, Probiotics, Complete feeds, High-throughput 16S rRNA, Antinutritional factors

## Abstract

**Abstract:**

Antinutritional factors present in complete feeds markedly diminish digestive and absorptive efficiency in animals, thereby affecting growth performance and resulting in economic losses. Liquid fermentation technology has been demonstrated to be an effective method of reducing antinutritional factors and enhancing the nutritional value of complete feeds. However, there is a lack of systematic research on the liquid fermentation and the screening of bacterial strains for use. In the present study, *Bacillus subtilis* and *Lactobacillus plantarum* characterized by excellent low-temperature tolerance, great enzyme activity, strong bacteriostatic capacity, and exceptional acid production, were evaluated for their suitability in a two-stage (aerobic followed by anaerobic) liquid fermentation process of complete pig feed. The results demonstrated that soybean antigenic protein and crude fiber underwent significant degradation, while crude protein and acid-soluble protein content exhibited significant increases in the feed following two-stage fermentation. Additionally, the accumulation of biogenic amines was inhibited to ensure the palatability of the feed. Furthermore, two-stage fermentation significantly enhanced the antioxidant and enzymatic activity of the feed. High-throughput 16S rRNA sequencing revealed an increased relative abundance of beneficial bacteria and a decreased abundance of pathogenic bacteria after fermentation. This study corroborated that a two-stage fermentation process could enhance the nutritional value, safety, and probiotic functionality of animal feeds. This finding provides a theoretical foundation for the development of functional fermented feeds and provides the necessary technical support for the practical application of liquid fermentation feeds.

**Key points:**

• *Developed a novel low-temperature two-stage liquid fermentation feed strategy using Bacillus subtilis 3–16 and Lactobacillus plantarum E5*

• *Significantly degraded antinutritional factors and biogenic amines, while increasing the crude protein, acid-soluble protein, enzymatic activity, and antioxidant capacity of complete pig feed*

• *Promoted beneficial microbiota dominance (e.g., Lactobacillus plantarum and Bacillus subtilis, while reducing pathogenic bacteria (e.g., Escherichia coli and Staphylococcus aureus)
*

**Supplementary Information:**

The online version contains supplementary material available at 10.1007/s00253-025-13633-9.

## Introduction

Complete feeds often contain legume, cottonseed meal, cereals, crude fiber, and animal-based protein, some of which may contain antinutritional factors (ANFs) that can hinder the healthy growth of animals (Wu et al. 2020). Soybean meal (SBM), for example, contains a variety of ANFs, including soybean antigenic proteins, phytic acid, and trypsin inhibitors (Cao et al. 2024a). These factors can impair digestion and nutrient absorption, cause gastrointestinal dysfunction and diarrhea, and in severe cases, lead to mortality and economic losses (Fu et al. 2007; Liu et al. 2025). Similarly, gluten in cereals may damage the intestinal barrier, leading to indigestion, disruption of gut microbiota, inflammation, and allergic reactions (Kohno et al. 2016). Crude fiber (CF) may affect palatability and reduce digestibility (Ratanpaul et al. 2019). Therefore, a major research focus for novel biological feeds is the use of probiotics in liquid fermentation, which produce organic acids and digestive enzymes and can achieve high nutrient utilization rates (Liao and Nyachoti 2017).

There are two main categories of fermentation: solid-state fermentation (SSF) and liquid-state fermentation (LSF). LSF has been shown to exhibit certain advantages over SSF, including shorter fermentation time (24–48 h), higher bacterial density (up to 10^9^ CFU/mL), and improved feed palatability (Matejčeková et al. 2019). These features make LSF particularly suitable for automated feeding systems in contemporary agricultural contexts.

Extensive studies have shown that LSF can reduce antigenic protein and CF levels to increase the length and depth of intestinal villi, thereby improving gut microbiota composition, enhancing immunity, and increasing meat yield (Missotten et al. 2015; Wang et al. 2021). However, the high moisture content of LSF makes it vulnerable to contamination by undesirable microorganisms. Moreover, maintaining key process parameters such as pH, dissolved oxygen, and temperature can be challenging (Singh et al. 2017; Su et al. 2018; Zhang et al. 2022). Failure to do so can easily lead to fermentation failure or reduce product quality. As a result, findings on LSF efficacy have been inconsistent; this was later contradicted by other researchers (Canibe and Jensen 2003), as they reported that finisher pigs fed liquid fermented feed showed significantly poorer growth performance compared to the control group.

These inconsistencies largely arise from the limitations of the single-stage fermentation. Under aerobic conditions, microbial metabolism accelerates the breakdown of proteins, carbohydrates, etc., but also promotes aerobic spoilage and increases dry matter loss (Tabacco et al. 2011). Additionally, during fermentation, free amino acids can be decarboxylated into biogenic amines (BAs), such as total volatile basic nitrogen (TVBN), putrescine (PUT), and spermidine (SPE), which affect feed odor and palatability and may cause allergic reactions or toxicity (Liao et al. 2024; Świder et al. 2024). Conversely, single anaerobic fermentation may be inadequate, as it can restrict the growth of certain aerobic or facultative anaerobic microorganisms (e.g., *Bacillus* sp., *Aspergillus* sp.), resulting in incomplete degradation of macromolecules such as proteins, nonstarch polysaccharides, and antigenic proteins. To address these issues, we adopted a two-stage fermentation strategy combining an aerobic phase and an anaerobic phase.

Furthermore, there is a lack of comprehensive evaluation systems for screening functional bacteria for pre-fermentation and for elucidating the synergistic metabolic mechanisms of mixed microbial communities (Du et al. 2024). In the study, we systematically screened fermentation-functional strains and developed an innovative two-stage fermentation model using *Bacillus* for aerobic fermentation and *Lactobacillus* for anaerobic fermentation. We investigated whether synergistic multi-strain fermentation could improve feed quality and optimize nutritional value, thereby improving pig health and growth.

## Materials and methods

### Chemicals and media

The culture media were purchased from Beijing Land Bridge Technology Co., Ltd. (Beijing, China); T-AOC test kit was purchased from Suzhou Keming Biotechnology Co., Ltd. (Jiangsu, China); 3M Petrifilm™ 6416 was purchased from 3M Company (USA); MDA test kit, Ethanol Content Assay Kit, Neutral Proteinase (NP) Activity Assay Kit, α-Amylase (α-AL) Activity Assay Kit, Cellulase (CL) Activity Assay Kit, and Phytase Activity Assay Kit were purchased from Beijing Solarbio Science&Technology Co., Ltd. (Beijing, China). All chemical reagents were obtained from Chengdu Chron Chemicals Co., Ltd (Sichuan, China). SBM, rice bran (RB), wheat, barley, wheat bran (WB), distillers dried grains with solubles (DDGS), and 883 complete pig feed (883-feed) were purchased from New Hope Liuhe Co., Ltd (Sichuan, China).

### Sample screening and isolation

A total of 71 *Bacillus* strains and 41 *Lactobacillus* strains were previously isolated from soil by the laboratory of New Hope Liuhe Co., Ltd (Sichuan, China). They are all preserved in the Sichuan Provincial Key Laboratory of New Hope Liuhe Co., Ltd. All strains involved in this study are available free of charge for research purposes upon request to the corresponding author. The strains of E5 (Deposit number: CGMCC NO.24432) can be obtained from the China General Microbiological Culture Collection Center (Beijing, China).

They were inoculated in LB and MRS and activated at 37 ℃ for 18 h (10^9^ CFU/mL). The activated strains were then inoculated into LB and MRS broth at 1% (v/v) (10⁷ CFU/mL) and incubated at 10 ℃, 15 ℃, and 20 ℃ for three successive passages. Cryotolerant strains were plated on skim milk agar, carboxymethylcellulose sodium (CMC-Na) agar, starch agar, calcium phytate agar, and Tween-80 agar, and incubated at 20 ℃ for 18 h. CMC-Na plates were stained with 2% Congo red, starch plates with iodine solution (Cao et al. 2024b), and calcium phytate plates with 0.04% bromocresol green. Clear or precipitated zones were recorded, and the diameters of enzymatic reaction zones (D) and colonies (d) were measured. Protease, cellulase, phytase, lipases, and amylase enzyme were determined using commercial diagnostic kits.

Screened strains were grown in LB and MRS at 20 ℃ for 18 h. The sterile supernatants were tested for inhibitory activity against *Escherichia coli*, *Salmonella*, *Pseudomonas aeruginosa*, *Streptococcus pneumoniae*, *Listeria monocytogenes*, *Staphylococcus aureus*, and *Erysipelothrix rhusiopathiae* (from the laboratory) using the Oxford cup method. The diameter of the inhibition zone was measured for each pathogen.

### Analysis of lysine utilization and preservative tolerance

The activated strains were inoculated into medium containing 0.1% Lysin and incubated at 20 ℃ for 24 h. Lysin content was quantified using an auto Amino Acid Analyzer (JLC-500/V2, Japan).

For preservative tolerance testing, strains were inoculated into LB or MRS containing 0.00%, 0.02%, 0.08%, 0.10%, and 0.08% benzoic acid and 0.1%, 0.15%, 0.2%, or 0.2% sodium benzoate, and incubated at 20 ℃ for 16 h. Optical density at 600nm was recorded, and survival rates were calculated as:

$$Survivalrate\%=OD600nm\left(A-C\right)/OD600nm\left(B-C\right)$$

Here, *A* is inoculated strains with mold inhibitor, *B* is inoculated strains without mold inhibitor, and *C* is LB/MRS without inoculated strains.

### Analysis of feedstuff fermentation

Feed ingredients (50 g) were mixed with 400 mL of distilled water in 1-L Erlenmeyer flasks. Each group was inoculated with 5% *Bacillus* (10⁷ CFU/mL) and aerobically fermented at 25 ℃ for 48 h. Samples were dried at 65 ℃ and analyzed for antigenic and alcohol-soluble proteins by SDS-PAGE, as well as for crude protein (CP), acid-soluble protein (ASP), crude fiber (CF), ash content (Ash), acid detergent fiber (ADF), crude ether extract (EE), and neutral detergent fiber (NDF), following established laboratory methods (Zhang et al. 2024).

For *Lactobacillus* fermentation, 883-feed (50 g) was mixed with 400 mL of distilled water, inoculated with 5% *Lactobacillus* (10⁷ CFU/mL), and anaerobically fermented at 25 ℃ for 48 h. pH was measured by pH Meter (Seven Compact S210, Shanghai), lactic acid, acetic acid, and formic acid were measured by Agilent 1260 Infinity II (Agilent Technologies, USA), and ethanol content was measured by diagnostic kits.

### Antagonistic relationship testing

Antagonism between strains was assessed following Cao et al. (2024b). *Bacillus* stains were inoculated onto the LB agar and incubated at 37 ℃ for 16 h. *Lactobacillus* stains were then streaked adjacent to *Bacillus* colonies and incubated in an anaerobic environment for 16 h. Growth inhibition was observed directly and documented.

### Screening of strain combinations for two-stage fermentation

Selected strains were cultured in LB/MRS at 37 ℃ for 12 h. For the aerobic stage, 100 g of 883-feed was mixed with 300 mL sterile distilled water in 1-L Erlenmeyer flasks, inoculated with 1% *Bacillus* (10⁷ CFU/mL), which were covered with a sterile membrane allowing only air exchange, and fermented at 25 ℃ for 12 h. Gross energy (GE) and dry matter (DM) loss rates were tested by an adiabatic bomb calorimeter (IKA C2000 Germany), after which the flasks were sealed and inoculated with 1% *Lactobacillus* (10⁷ CFU/mL); then the membrane was removed, and the flasks were sealed with rubber stoppers for anaerobic fermentation at 25 ℃ for another 12 h. All samples were prepared in triplicates. Final GE and DM-loss rates were then measured. The optimal strain combination was identified based on performance and taxonomic identification (Gao et al. 2020).

### Two-stage fermentation trial

Three treatments were prepared of N-group, TN-group, and TS-group.

N-group: 100 g of 883-feed was mixed with 300 mL of sterile distilled water in 1-L Erlenmeyer flasks, inoculated with 5% sterile distilled water for aerobic fermentation (30 ℃, 0–12 h), followed by inoculation with 1% sterile distilled water for anaerobic fermentation (30 ℃, 12–24 h).

TN-group: 100 g of 883-feed was mixed with 300 mL of sterile distilled water, inoculated with 5% *Bacillus* (10⁷ CFU/mL) for aerobic fermentation (30 ℃, 0–12 h), followed by inoculation with 1% *Lactobacillus* (10⁷ CFU/mL) for anaerobic fermentation (30 ℃, 12–24 h).

TS-group: 100 g of 883-feed was mixed with 290 mL of sterile distilled water and 10 mL of 8% benzoic acid, inoculated with 5% *Bacillus* (10⁷ CFU/mL) for aerobic fermentation (30 ℃, 0–12 h), followed by inoculation with 1% *Lactobacillus* (10⁷ CFU/mL) for anaerobic fermentation (30 ℃, 12–24 h).

The operation of aerobic and anaerobic fermentation was consistent with those described above. All samples were prepared in triplicates. Samples were collected at 0, 6, 12, 18, and 24 h for microbial, biochemical, and molecular analyses. The remaining samples were dried at 65 ℃ for chemical analysis and SDS-PAGE.

### SDS-PAGE analysis

Proteins analysis for SDS-PAGE was consistent with that described above.

### Analysis of antioxidant capacity, enzymatic activity, BAs, and nutrient content

The total antioxidant capacity (T-AOC) and malondialdehyde (MDA) concentrations of fermentation samples were determined at 0 h, 12 h, and 24 h using commercial kits. Protease, cellulase, and amylase activities were also assayed at these time points using commercial kits. Chemical analyses of CP, ASP, CF, ADF, GE, TAN, DM-loss rate were consistent with those described above. The separation and quantification of BAs were carried out according to the liquid chromatography–tandem mass spectrometry method by Agilent 1260 Infinity II (Agilent Technologies, USA) (Chen et al. 2022a).

### Analysis of microbial diversity

Samples from each group were collected at 0, 6, 12, 18, and 24 h, immediately frozen in liquid nitrogen, and stored in sterile EP tubes at −80 °C until analysis. Frozen samples were transported on dry ice to the laboratory for DNA extraction. The V4 region of the bacterial 16S rDNA was amplified by polymerase chain reaction (PCR). Illumina NovaSeq 6000 high-throughput sequencing was performed by Novogene Bio-informatics Co., Ltd. (Beijing, China). The data were analyzed on the online platform of Novo Magic Cloud Platform. Based on the annotation information of species, the OTUs that cannot be annotated to the boundary level and their contained sequences were removed and the diversity of bacteria in the samples at the family and genus levels was analyzed. Spearman’s correlation analysis was conducted to analyze the correlations among nutritional indicators (CP, CF, ASP, TAN, pH, enzyme activity), BAs (TVBN, PUT, SPE), antioxidant capacity (MDA, T-AOC), and microbiota of fermentation feed.

### Statistical analysis

All data were analyzed using SPSS 20.0 software (SAS Inc., Chicago, IL, USA). One-way ANOVA followed by Duncan’s multiple range test was used to assess the differences among means. A *P*-value < 0.05 was considered statistically significant. GraphPad Prism 10 (GraphPad Software, San Diego, CA, USA) was used for graphical presentation of the results.

## Results

### Screening of fermentation-functional bacteria

**Fig. 1:**
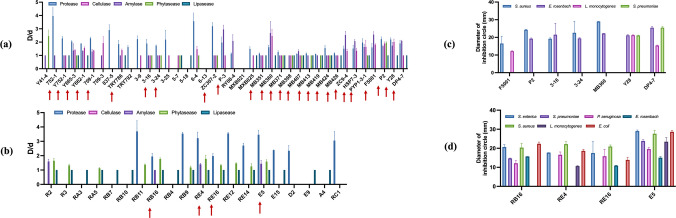
Enzymatic activity and bacteriostatic capacity of functional bacteria. **a** Enzymatic activity of *Bacillus*. **b** Enzymatic activity of *Lactobacillus.* **c** Bacteriostatic capacity of *Bacillus.* **d** Bacteriostatic capacity of *Lactobacillus.* Data are means with standard error bars (*n* = 3). Red arrows indicate the strains that displayed a minimum of 4 enzyme activities. “D” means the diameters of enzymatic reaction zones, colonies; “d” means the diameters of colonies

We isolated 39 *Bacillus* and 22 *Lactobacillus* strains capable of growth at 20 ℃. Among these 61 functional strains, 23 *Bacillus* (Fig. 1a) and 4 *Lactobacillus* (Fig. 1b) exhibited four or more distinct enzymatic activities.

Seven *Bacillus* strains, P2 (*Bacillus subtilis*), F5001 (*Bacillus belieriensis*), MB360 (*Bacillus subtilis*), 3–16 (*Bacillus subtilis*), 3–24 (*Bacillus licheniformis*), DP4-7 (*Bacillus amyloliquefaciens*), and Y28 (*Bacillus subtilis*), displayed antibacterial activity. Specifically, F5001, P2, 3–16, 3–24, and MB360 showed strong inhibition of *S. aureus*, while all *Bacillus* strains except F5001 inhibited *E. rhusiopathiae* (Fig. 1c). All four *Lactobacillus plantarum* strains, E5, RB16, RE4, and RE16, exhibited antimicrobial capacity, with E5 being the most effective (Fig. 1d).

In conclusion, 7 *Bacillus* strains and 4 *Lactobacillus plantarum* strains were selected for further study based on provided enzymatic activities and bacteriostatic capacities.

### Enzyme activity of the functional strains

**Fig. 2:**
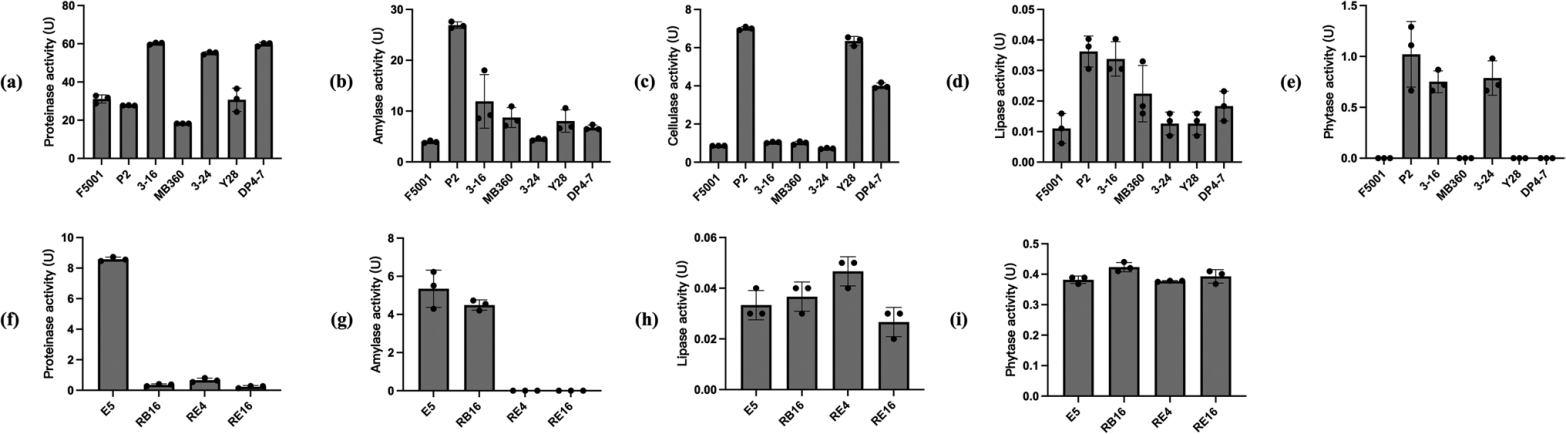
Enzymatic activity of functional bacteria. **a**–**e** Protease, amylase, cellulase, lipase, and phytase activity of *Bacillus*. **f**–**i** Protease, amylase, lipase and phytase activity of *Lactobacillus.* Data are means with standard error bars (*n* = 3)

Strains 3–16 and DP4-7 had the highest protease activities (Fig. 2a), while P2 had the highest amylase (Fig. 2b) and cellulase activities (Fig. 2c). However, *Lactobacillus* strains showed generally low amylase, lipase, and phytase activities (Fig. 2g–i), with E5 having the highest protease activity (Fig. 2f), though still lower than that of *Bacillus* strains.

### Lysine utilization and preservative tolerance

**Fig. 3:**
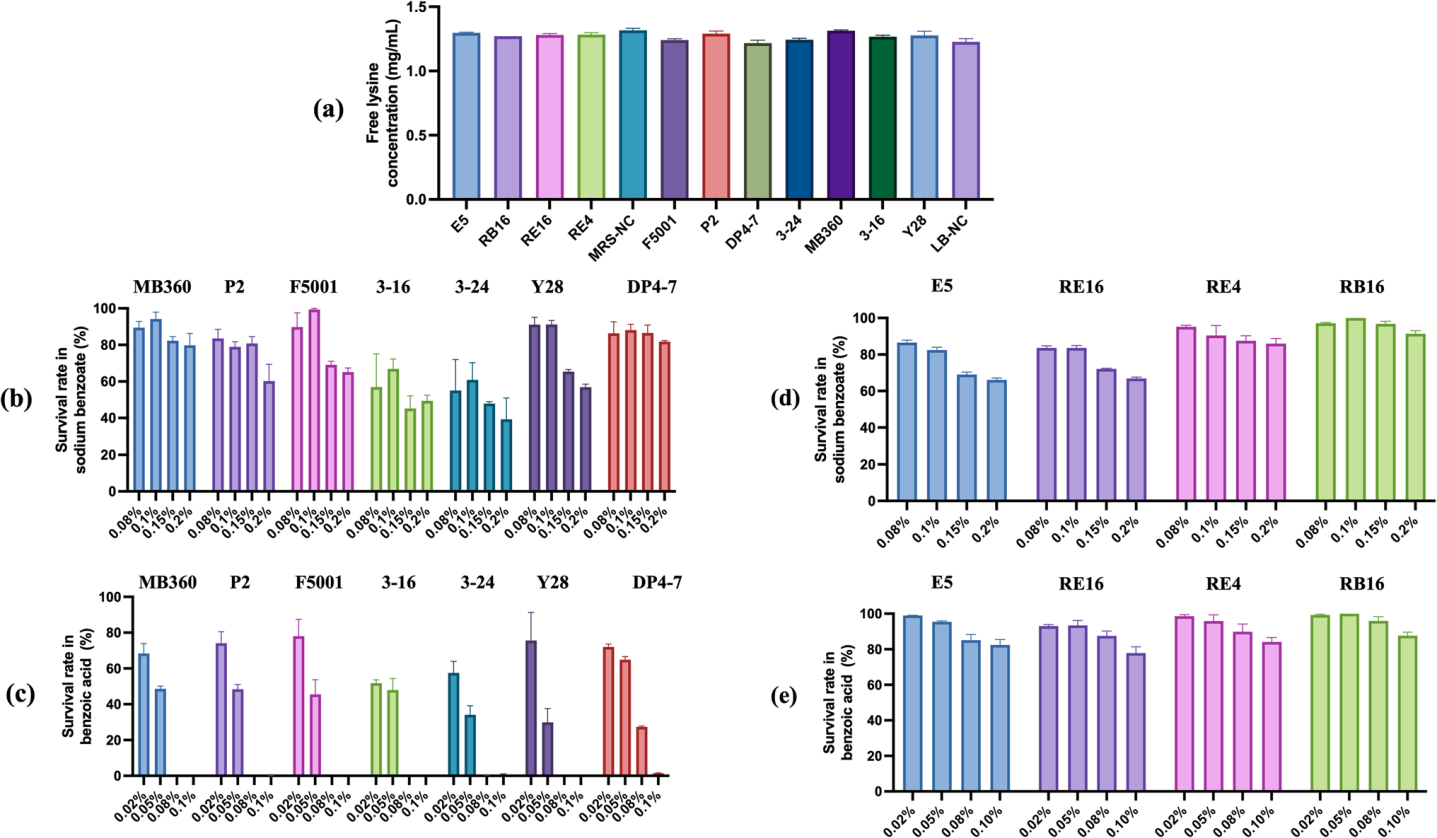
Evaluation of the functional bacteria’s lysine utilization and preservative tolerance. **a** Lysine concentration in the culture medium of *Bacillus* and *Lactobacillus*. **b** Survival rate of *Bacillus* in different sodium benzoate concentrations*.* **c** Survival rate of *Bacillus* in different benzoic acid concentrations*.* **d** Survival rate of *Lactobacillus* in different sodium benzoate concentrations*.* **e** Survival rate of *Lactobacillus* in different benzoic acid concentrations*.* Data are means with standard error bars (*n* = 3)

Overall, lysine utilization by functional strains was low. RB16 showed the highest utilization at 3.78% (Fig. 3a). The *Bacillus* strains generally tolerated the sodium benzoate well, with a survival rate of over 50% at 0.2% (Fig. 3b). However, *Bacillus* strains were sensitive to benzoic acid. There was no growth at 0.08%, and DP4-7 was completely inhibited at 0.1% (Fig. 3c). *Lactobacillus* strains tolerated both preservatives well (Fig. 3d, e), which supports their potential for industrial fermentation where preservatives are present.

### Fermentation capacity of functional bacteria

Composition changes in various substrates after fermentation are shown in Table 1. For SBM fermentation, *Bacillus* fermentation significantly increased CP, but had little effect on CF. For DDGS fermentation, CP increased in all groups, Ash increased in DP4-7, F5001, and P2 groups; EE decreased in all groups except the F5001 and 3–24 groups. Wheat flour and barley flour fermentation significantly increased CP, reduced ADF and NDF. WB fermentation increased CF in all groups.

**Table 1:** Compositional analysis of feed ingredients after fermentation by *Bacillus*

Feed	Parameters	Feed ingredients	Y28	DP4-7	MB360	F5001	P2	3–16	3–24
SBM	CP	51.35 ± 0.77	56.24 ± 1.01*	54.02 ± 0.10	55.44 ± 0.57*	56.03 ± 0.09*	55.30 ± 1.05*	56.75 ± 0.40*	56.14 ± 0.08*
	CF	6.68 ± 0.22	9.18 ± 0.16*	8.65 ± 0.11	9.40 ± 0.02*	10.11 ± 0.12*	9.29 ± 0.27*	6.74 ± 0.09	10.15 ± 0.09*
RB	CP	17.00 ± 0.03	21.77 ± 0.04*	18.39 ± 0.23*	18.25 ± 0.22*	17.57 ± 0.12	19.88 ± 0.13*	18.8 ± 0.77*	19.58 ± 0.08*
	CF	9.55 ± 0.03	9.77 ± 0.04	10.27 ± 0.23*	10.32 ± 0.22*	10.83 ± 0.12*	11.24 ± 0.13*	10.93 ± 0.77*	12.13 ± 0.08*
	ADF	9.61 ± 1.50	11.70 ± 0.47*	10.25 ± 0.07	11.74 ± 0.26*	11.99 ± 0.21*	12.44 ± 0.21*	13.42 ± 0.13*	14.00 ± 1.18*
	NDF	22.34 ± 1.79	21.17 ± 1.03	20.85 ± 0.34*	21.98 ± 0.50	20.66 ± 0.78*	22.99 ± 0.32	23.51 ± 0.33	22.31 ± 0.59
DDGS	CP	29.05 ± 0.16	31.22 ± 0.56*	31.08 ± 0.23*	31.67 ± 0.38*	26.88 ± 0.43*	30.03 ± 0.59	30.59 ± 0.79	27.26 ± 3.17
	CF	7.00 ± 0.09	6.54 ± 0.18	6.03 ± 0.65	7.31 ± 1.09	6.81 ± 0.62	6.46 ± 0.38	7.08 ± 0.29	6.71 ± 0.19
	EE	14.32 ± 0.16	12.19 ± 0.38*	10.71 ± 0.23*	13.84 ± 0.42	14.12 ± 0.35	11.75 ± 0.27*	12.75 ± 0.23	12.66 ± 3.13
	Ash	5.64 ± 0.04	5.87 ± 0.20	7.59 ± 0.32	5.02 ± 0.35	7.91 ± 0.83*	7.33 ± 0.26	5.66 ± 0.11	8.32 ± 0.55*
Wheat	CP	15.5 ± 0.30	16.87 ± 0.38	16.79 ± 0.58	17.19 ± 0.07	17.26 ± 0.30	20.46 ± 5.85*	16.96 ± 0.21	17.53 ± 0.15
	CF	2.30 ± 0.01	2.40 ± 0.08	2.65 ± 0.24	2.31 ± 0.01	2.54 ± 0.11	2.25 ± 0.04	2.43 ± 0.02	2.27 ± 0.03
	ADF	3.29 ± 0.04	2.68 ± 0.06	2.77 ± 0.05	2.68 ± 0.03	2.72 ± 0.02	2.51 ± 0.13	2.57 ± 0.13	2.50 ± 0.17
	NDF	11.00 ± 0.76	7.04 ± 0.01*	8.16 ± 0.12*	6.98 ± 0.02*	7.11 ± 0.07*	6.87 ± 0.74*	6.93 ± 0.13*	6.80 ± 0.35*
Barley	CP	11.16 ± 0.06*	12.82 ± 0.01*	12.66 ± 0.36*	12.59 ± 0.26*	12.52 ± 0.36*	12.18 ± 0.10	13.24 ± 0.42*	13.04 ± 0.72*
	CF	5.52 ± 0.06	5.66 ± 0.26	6.18 ± 0.34	5.87 ± 0.56	5.82 ± 0.12	5.76 ± 0.16	5.61 ± 0.18	4.97 ± 0.04
	ADF	5.70 ± 0.02	4.97 ± 0.33	5.55 ± 0.44	5.66 ± 0.49	5.51 ± 0.09	5.55 ± 0.16	5.15 ± 0.15	5.01 ± 0.02
	NDF	18.55 ± 0.20	11.83 ± 0.51*	13.84 ± 1.06*	14.23 ± 1.85*	13.68 ± 0.32*	13.87 ± 0.44*	12.9 ± 0.24*	12.27 ± 0.59*
WB	CP	16.91 ± 0.02	20.17 ± 0.24*	19.74 ± 1.05*	20.63 ± 0.24*	20.55 ± 0.07*	20.29 ± 0.18*	19.65 ± 0.13*	20.02 ± 0.01*
	CF	11.85 ± 0.02*	13.79 ± 0.26*	13.93 ± 0.47*	13.65 ± 0.06*	14.51 ± 0.21*	14.86 ± 0.25*	13.57 ± 0.19*	13.42 ± 0.07
	ADF	12.67 ± 0.39	15.48 ± 0.41*	16.63 ± 0.27*	15.34 ± 0.16*	16.15 ± 0.34*	17.47 ± 0.73*	13.83 ± 0.18	14.94 ± 0.55*
	NDF	40.91 ± 0.09	42.50 ± 2.46	47.91 ± 0.89*	43.26 ± 0.81*	44.76 ± 1.90*	49.77 ± 1.85*	40.07 ± 0.10	42.91 ± 0.12*

Values are mean ± SD (*n* = 3). * indicates a significant difference compared to the feed ingredients (* *P* < 0.05)

**Fig. 4:**
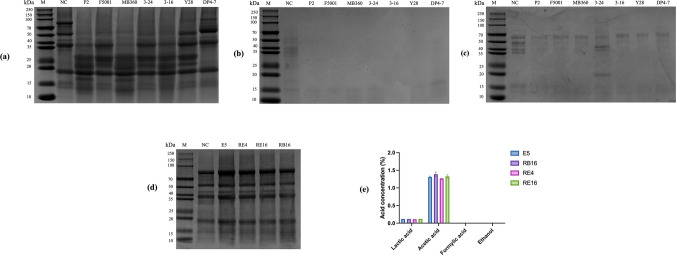
SDS-PAGE protein analysis of *Bacillus* and analysis of acid-producing species of *Lactobacillus.* **a** Protein profiling of SBM using SDS-PAGE over a period of 48 h under aerobic environment by *Bacillus.* **b** Protein profiling of wheat using SDS-PAGE over a period of 48 h under aerobic environment by *Bacillus.* **c** Protein profiling of barley using SDS-PAGE over a period of 48 h under aerobic environment by *Bacillus.* **d** Protein profiling of SBM using SDS-PAGE over a period of 48 h under aerobic environment by *Lactobacillus*. **e** Concentration of acids after fermentation of 883-feed by *Lactobacillus*. Data are means with standard error bars (*n* = 3)

SDS-PAGE analysis showed that after 48-h SBM fermentation, 72, 68, and 34 kDa proteins were degraded mainly into fragments less than 25 kDa, with P2, MB360, and 3–16 being most effective (Fig. 4a). In wheat, alcohol-soluble proteins of 30–45 and 65–80 kDa were degraded (Fig. 4b). with barley showing greater degradation in the same range (Fig. 4c). *Lactobacillus* strains were largely ineffective in degrading antigenic proteins (Fig. 4d). In 883-feed fermentation, *Lactobacillus* produced lactic and acetic acid but negligible ethanol or formic acid (Fig. 4e).

### Antagonistic relationship of bacteria

**Fig. 5:**
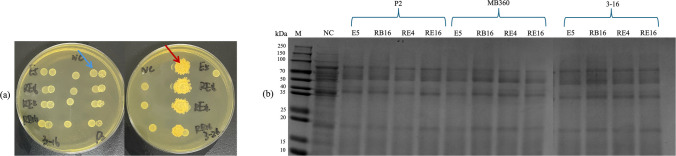
Schematic representation of antagonistic relationships and SDS-PAGE protein analysis of two-stage fermentation by combined strains. **a** Schematic representation of antagonistic relationships. The presence of blue arrows indicates that the two strains are not antagonistic, while red arrows indicate that they are antagonistic. **b** SDS-PAGE protein analysis. The strains above the curly brackets were subjected to a combined two-stage fermentation (aerobic first) with the strain below the curly brackets (anaerobic later)

Supplemental Table S1 shows the antagonistic relationships between *Bacillus* and *Lactobacillus*. MB360, 3–16, P2, and DP4-7 showed no significant antagonism (the colony morphology of *Lactobacillus* is circular). While 3–24 and Y28 exhibited strong antagonism and were excluded from further trials (Fig. 5a), the colony morphology of *Lactobacillus* exhibits a crescent shape.

### Screening of combined strains in two-stage fermentation

Twelve combinations were tested. 3–16 and E5 produced the lowest pH, lowest loss rate, and high GE after fermentation (Table 2), and effectively degraded 72, 52, 34, and 19 kDa antigenic proteins (Fig. 5b). The combination of 3–16 and E5 was selected for subsequent trials (E5 patent number: ZL 202211081870.7, NCBI accession number: PX36230, 3–16 NCBI accession number: PX149098).

**Table 2:** Determination of pH, DM-loss rate, and GE in fermentation of combined strains

Groups		pH	DM-loss rate (%)	GE (cal/g)
MB360	E5	4.73 ± 0.04*	6.89 ± 0.38	4283.00 ± 8.00
	RB16	4.88 ± 0.02	5.72 ± 0.75	4314.50 ± 31.50*
	RE4	4.86 ± 0.01*	6.62 ± 0.98	4328.50 ± 28.50*
	RE16	4.74 ± 0.10*	6.34 ± 0.57	4335.50 ± 14.50*
3–16	E5	4.72 ± 0.02*	5.11 ± 0.29	4307.50 ± 20.50*
	RB16	4.88 ± 0.01	6.34 ± 0.23	4324.00 ± 9.00*
	RE4	4.84 ± 0.02*	5.95 ± 0.75	4289.50 ± 7.50*
	RE16	4.89 ± 0.01	6.36 ± 0.29	4313.00 ± 21.00*
P2	E5	4.83 ± 0.01*	6.52 ± 0.31	4262.00 ± 42.00
	RB16	4.73 ± 0.01*	5.21 ± 0.40	4277.00 ± 34.00
	RE4	4.72 ± 0.01*	6.82 ± 0.15	4280.00 ± 23.00
	RE16	4.89 ± 0.02	5.19 ± 0.50	4248.00 ± 19.00
883-feed		4.98 ± 0.01	5.80 ± 0.27	4230.33 ± 2.89

Values are mean ± SD (*n* = 3). * indicates a significant difference compared to the 883-feed (*P < 0.05)

### Nutrient composition changes during two-stage fermentation

**Fig. 6:**
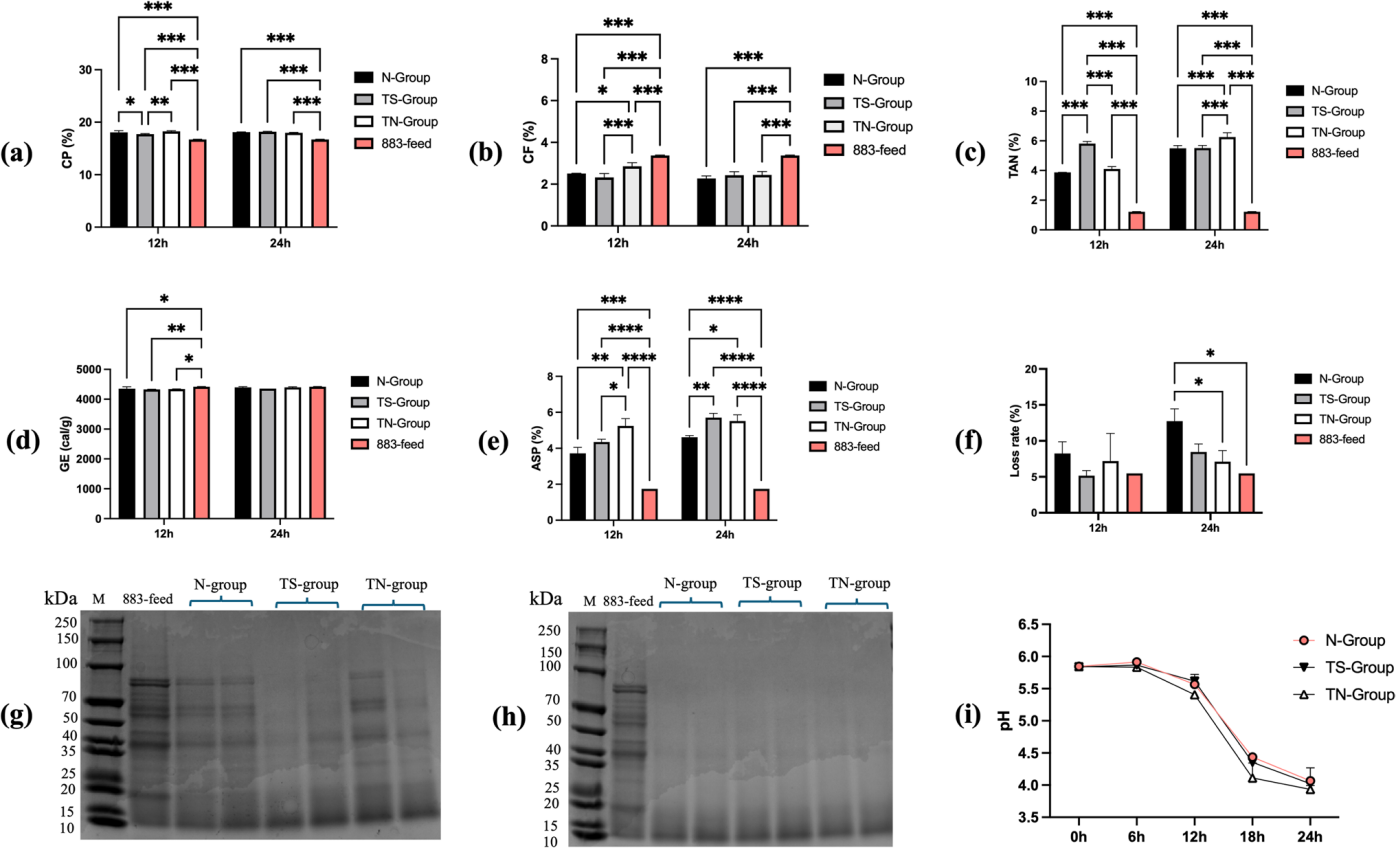
Results of pH and compositional changes in two-stage fermentation. **a** The changes in CP concentration of 883-feed in two-stage fermentation. **b** The changes in CF concentration of 883-feed in two-stage fermentation. **c** The changes in TAN concentration of 883-feed in two-stage fermentation. **d** The changes in GE of 883-feed in two-stage fermentation. **e** The changes in ASP concentration of 883-feed in two-stage fermentation. **f** The changes in loss rate of 883-feed in two-stage fermentation. **g** Protein profiling of 883-feed using SDS-PAGE over a period of 12 h under aerobic environment by *Bacillus.* **h** Protein profiling of 883-feed using SDS-PAGE over a period of two-stage fermentation*.* **i** The changes in pH of 883-feed in two-stage fermentation. Data are means with standard error bars (*n* = 3). Significant differences are indicated with asterisks (*P < 0.05, **P < 0.01, ***P < 0.005)

Through Fig. 6, we can observe the changes in the nutritional components of the 883-feed during the fermentation process. CP increased significantly in all fermented groups (Fig. 6a). CF content decreased significantly (Fig. 6b). TAN increased after fermentation; TS-group was significantly higher than the N-group at 12 h and TS-group and TN-group was significantly higher than that of the N-group at 24 h (Fig. 6c). GE remained unchanged (Fig. 6d). ASP content increased significantly, and TN-group was significantly higher than N-group at 12 h; TS-group and TN-group had significantly higher than N-group at 24 h (Fig. 6e). Loss rates increased with N-group higher than TN-group by 24 h (Fig. 6f). Antigenic protein degradation was higher in the TS- and TN-groups than in the N-group, after 12 h. This was particularly evident in the TS-group, where the proteins had almost completely degraded into small peptides (Fig. 6g). However, after 24 h, antigenic proteins had almost completely degraded in all groups (Fig. 6h). In all groups, pH increased slightly during the early aerobic phase and sharply after *Lactobacillus* inoculation. Final pH was 4.3 in the TS-group and TN-group and 4.5 in N-group (Fig. 6i).

### Biogenic amine changes in a two-stage fermentation

**Fig. 7:**
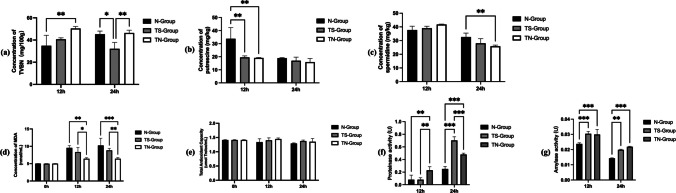
Results of BAs concentration, antioxidant properties and enzyme activity in two-stage fermentation. **a** The changes in TVBN concentration of 883-feed in two-stage fermentation. **b** The changes in PUT concentration of 883-feed in two-stage fermentation. **c** The changes in SPE concentration of 883-feed in two-stage fermentation. **d** The changes in MDA concentration of 883-feed in two-stage fermentation. **e** The changes in T-AOC capacity of 883-feed in two-stage fermentation. **f** The changes in protease activity of 883-feed in two-stage fermentation. **g** The changes in amylase activity of 883-feed in two-stage fermentation. Data are means with standard error bars (*n* = 3). Significant differences are indicated with asterisks (*P < 0.05, **P < 0.01, ***P < 0.005)

TVBN increased in N-group throughout fermentation and was significantly higher than TS-group at 24 h (Fig. 7a). PUT was higher in N-group than TS-group and TN-group at 12 h, but differences disappeared at 24 h, with N-group still highest (Fig. 7b). SPE content did not differ at 24 h, but was highest in N-group at 24 h, significantly exceeding TN-group (Fig. 7c).

### Antioxidant and enzymatic activities in two-stage fermentation

During fermentation, MDA levels in N-group were significantly higher than TN-group at 12 and 24 h (Fig. 7d). However, there was no significant difference in T-AOC (Fig. 7e). At 24 h, TS-group had the highest protease activity and TN-group had the highest amylase activity, both significantly greater than N-groups (Fig. 7f, g).

### Microbial community dynamics in two-stage fermentation

**Fig. 8:**
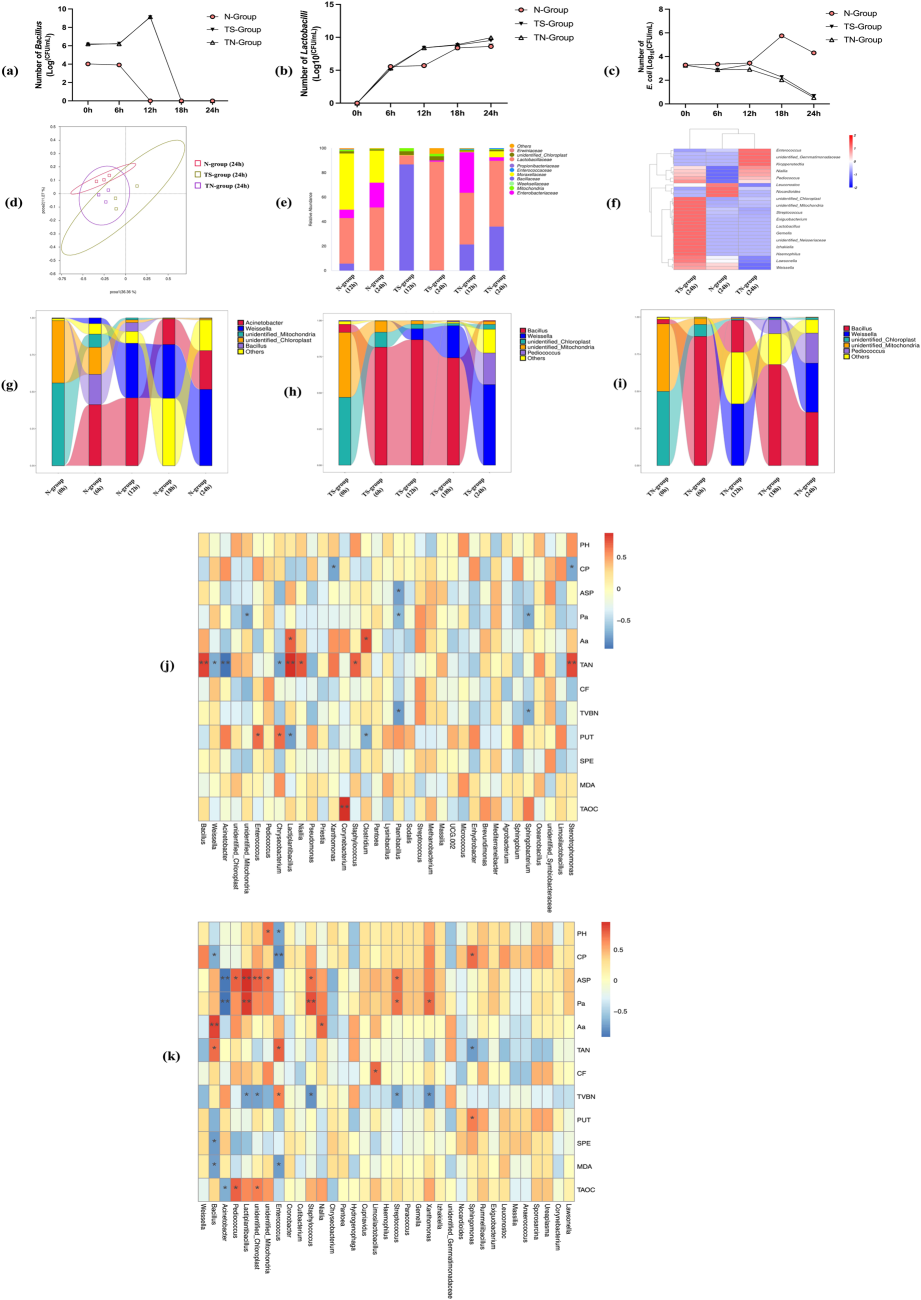
Microbial analysis during the two-stage fermentation. **a** The number of *Bacillus* in two-stage fermentation. **b** The number of *Lactobacillus* in two-stage fermentation. **c** The number of *E. coli* in two-stage fermentation. **d** PCoA plots of different treatment groups after 24-h fermentation. **e** The family level composition of the microflora in different treatment groups. **f** Heatmap analysis of different treatment groups. The samples with the highest and lowest bacterial levels are in red and blue, respectively. **g** The Sankey diagram of N-group in 12 h and 24 h. **h** The Sankey diagram of TS-group in 12 h and 24 h. **i** The Sankey diagram of TN-group in 12 h and 24 h. **j** Spearman’s correlation analysis of samples from 883-feed that was fermented for 12 h. **k** Spearman’s correlation analysis of samples from 883-feed that was fermented for 24 h. Pa means protease activity, Aa means amylase activity. Grids in orange indicate positive correlations, while grids in blue indicate negative correlations. The color-coding scale indicates the correlation analysis value from the heatmap, and the deeper blue or orange indicates higher correlation values. Data are means with standard error bars (*n* = 3). * indicates a significant difference (*P < 0.05, **P < 0.01)

After *Bacillus* inoculation, TS- and TN-groups had larger *Bacillus* counts than N-group (Fig. 8a). N-group harbored more diverse bacterial species, including *E. coli* and *S. aureus*, while *Lactobacillus* counts increased rapidly after inoculation in TN- and TS-groups, surpassing N-group (Fig. 8b)*.* Subsequently, the number stabilized and eventually exceeded that in the N-group. Besides, only *Lactobacillus* was detected in the TN- and TS-groups, whereas anaerobic strains were more abundant in the N-group. In terms of pathogenic bacteria, *E. coli* counts peaked at 18 h in N-group but declined steadily in TN- and TS-groups (Fig. 8c).

To further understand the variance in the bacterial community structure of 883-feed during fermentation, the principal co-ordinates analysis (PCoA) was performed, and PCoA showed distinct OTU profiles among N-, TS-, and TN-group (Fig. 8d). The changes in family level bacterial communities in 883-feed after fermentation are shown in Fig. 8e. The major family in 883-feed of N-group was *Moraxellaceae*, *Enterobacteriaceae*, and *Lactobacillaceae*. The major family in 883-feed of TS-group was *Lactobacillaceae* and the major family in 883-feed of TN-group was *Lactobacillaceae* and *Bacillus.* Heatmap representation of the clustered distant matrix data was used to visualize the microbial composition of different locations of samples by 16S rRNA sequencing. As shown in Fig. 8f, the microbial communities of the N-group were different compared with those of the TS-group and TN-group at the genus level.

To investigate the microbiota composition in different spatial locations, we calculated the relative abundance of different genus shown in a Sankey diagram (Fig. 8g–i). In the figures, the lines of different colors represent different bacterial genus, and the height of the rectangles indicates the relative abundance in the genus. At the genus level, we found that N-group has a more complex flora variation, and the abundance of *Acinetobacter* remained high after anaerobic fermentation. The pattern of change in flora in the TS- and TN-groups was consistent with the timing of probiotic addition throughout the experiment. The highest abundance of *Bacillus* was observed after 6 h of the aerobic phase. After subsequent anaerobic fermentation, the abundance of *Pediococcus* and *Weissella* in the TS-group stayed high, while the abundance of *Bacillus* declined. At the end of fermentation, the abundance of *Bacillus* in the TN-group stayed high, while the abundance of *Acinetobacter* decreased. At the genus level, N-group was dominated by *Moraxellaceae*, *Enterobacteriaceae*, and *Lactobacillaceae*, TS-group by *Lactobacillaceae*, and TN-group by *Lactobacillaceae* and *Bacillaceae*. Heatmaps and Sankey diagrams confirmed shifts toward probiotic dominance and pathogen reduction in TS- and TN-groups.

### Spearman’s correlation analysis

We assessed the relationships between pH, nutrient composition, enzymatic activity, BAs, and antioxidant activity with strains using Spearman’s correlation analysis. At 12 h, TAN was positively correlated with *Bacillus* and *Laciplantibacillus*, and TVBN was negatively correlated with *Paenibacillus* and *Sphingobacterium*. PUT correlated positively with *Enterococcus* and *Chryseobacterium* but negatively with *Laciplantibacillus* and *Clostridium*. T-AOC was positively correlated with *Corynebacterium* (Fig. 8j). At 24 h, pH was negatively correlated with *Enterococcus*. ASP was positively correlated with *Laciplantibacillus* and *Pediococcus*, and negatively with *Acinetobacter*. Enzymatic activity was positively correlated with *Bacillus* and *Laciplantibacillus*, and negatively with *Acinetobacter*. TVBN was negatively correlated with *Laciplantibacillus* and *Staphylococcus*, and positively with *Enterococcus*. *Bacillus* was negatively correlated with spermidine and MDA. T-AOC was negatively correlated with *Acinetobacter* and positively with *Pediococcus* (Fig. 8k).

## Discussion

In livestock production, the nutritional value of feed directly influences animal growth and production efficiency. Numerous studies have demonstrated that additional processing of feed prior to feeding—through physical, chemical, or biological methods—can effectively enhance its nutritional properties. For instance, thermal treatment can degrade ANFs and improve protein digestibility (Ruiz-Zambrano et al. 2024), while fermentation can break down cellulose structures and increase probiotic content, thereby promoting gut health (Miraalami et al. 2025). Physical processing methods such as micritization and extrusion can also enhance starch gelatinization and energy utilization efficiency (Gonthier et al. 2004; Liermann et al. 2019). Furthermore, co-fermented feeds have been shown to confer substantial digestive, immune, and intestinal benefits, ultimately improving animal growth performance (Liu et al. 2024; Su et al. 2022b). In this study, we employed a two-stage fermentation process—an aerobic phase followed by an anaerobic phase to improve the nutritional value of pig feed.

Conventional fermentation typically requires additional heating to 30–37 ℃, which substantially increases the time and energy costs (Woo et al. 2020). Therefore, this study firstly considered the fermentation characteristics of probiotics under low-temperature conditions. We found that the selected strains maintained metabolic activities at 20 ℃. Our *Bacillus* strains exhibited high protease activities (59.74 U), compared to the *B. velezensis* Z-1(16.92 U) (Lu et al. 2022), and high-yield protease bacteria from Daqu of ZhangGong Laojiu (75–100 U) (Liu et al. 2022); this may be associated with the regulation of multiple metabolic pathways (Yang et al. 2023). Bioactive enzymes play a crucial role in feed fermentation (Yang et al. 2025), and enzyme activity is widely used as a key criterion for selecting functional fermentation strains (Borah et al. 2025; Wongputtisin et al. 2012). Notably, low-temperature incubation did not substantially reduce the bacteriostatic activity of probiotics, possibly due to the production of antimicrobial peptides by *Bacillus* and the low-pH environment generated by *Lactiplantibacillus* via organic acid production (Kiousi et al. 2023).

We also examined lysine utilization and preservative tolerance. Lysine is the first limiting amino acid in pig and poultry diets, and its content directly affects biological value (Goethals et al. 2025). The selected strains did not significantly metabolize lysine (residual > 95%), avoiding lysine depletion during fermentation. Researchers (Lau et al. 2022) have reported near-complete lysine loss during natural fermentation, whereas probiotic-inoculated fermentation reduced this loss to 10%. Thus, screening for strains that do not utilize lysine is essential. Furthermore, most strains displayed strong tolerance to benzoic acid and sodium benzoate, which are effective antimold agents. This indicated that they could remain metabolically active in feed containing preservatives, ensuring process stability (Pérez-Díaz et al. 2022; Zhang et al. 2020). The selected strains significantly improved the nutritional profile of soybean meal, wheat, barley, and DDGS. Fermentation increased CP content and reduced EE, likely due to microbial proliferation and associated protease and lipase activity. Antigenic proteins in SBM and alcohol-soluble proteins in cereals were effectively degraded, probably through enhanced protease activity and acid-mediated protein modification (Park et al. 2020). Such degradation improves feed digestibility and reduces intestinal allergic responses in young animal (Barratt et al. 1978). These results are consistent with the observed enzymatic activity profiles of the selected strains.

Microbial interactions are critical during fermentation. In the aerobic stage, *Bacillus* influences the subsequent growth of *Lactobacillus* and the biochemical composition of the fermentation (García et al. 2019). We confirmed that selected *Bacillus* strains do not inhibit *Lactiplantibacillus* growth*.* The optimal pairing identified *B. subtilis* 3–16 for the aerobic phase and *L. plantarum* E5 for the anaerobic phase.

Therefore, we conducted a follow-up study to evaluate the effect of the 3–16 and E5 combination on 883-feed after two-stage fermentation.

Two-stage fermentation with this combination significantly increased CP and ASP levels, while reducing CF. In the aerobic phase, *Bacillus* effectively degraded the plant cell wall structure by secreting protease and cellulase enzymes in abundance, releasing the encapsulated protein fractions (Chen et al. 2022b). The anaerobic phase further hydrolyzed proteins into small peptides and amino acids, increasing ASP (Sun et al. 2023). These findings are consistent with previous studies (Shi et al. 2017; Yao et al. 2021). Importantly, fermentation loss rates were lower than in the natural fermentation of N-group; this is similar to the previous research (Xu et al. 2024).

SDS-PAGE analysis confirmed the degradation of major antigenic proteins. Such proteins can trigger hypersensitivity reactions, intestinal barrier disruption, villus atrophy, and impaired digestion (Li et al. 2024; Wang et al. 2023). Their removal reduces allergic responses in weaned piglets and calves. Previous studies have shown that *B. amyloliquefaciens* LX-6 can achieve 57–78% degradation of glycinin and β-conglycinin (Huang et al. 2023; Milmine et al. 2025) Our results reinforce the superior efficiency of microbial multi-enzyme systems compared to heat or single-enzyme treatments.

Oxidative stability is another important quality parameter. MDA is a marker of lipid peroxidation, while T-AOC reflects antioxidant potential (Song and Shurson 2013). Although MDA levels increased toward the end of fermentation, they remained significantly lower in the two-stage fermentation group than in the control, consistent with other findings (Su et al. 2022a; Wu et al. 2024). Probiotic-associated antioxidant effects may derive from extracellular polysaccharides, vitamins, superoxide dismutase, catalase, and metal-iron chelation (Echegaray et al. 2023; Wang et al. 2017). Protease activity increased by 64.29% (TS-group), and amylase activity increased by 28.3% (TS-group), following two-stage fermentation, that lead to improved feed digestibility (Gao et al. 2020). Four BAs, such as histamine, tyramine, putrescine, are harmful nitrogenous compounds formed via microbial amino acid decarboxylation (Silla Santos 1996). They can reduce feed intake, impair nutrient absorption, and cause toxicity (Ruiz-Capillas and Herrero 2019). In our study, BAs levels remained low, with total volatile basic nitrogen significantly reduced in two-stage fermentation compared to N-group. This likely reflects both competitive inhibition of BAs-producing microbes and pH reduction by lactic acid bacteria (İncili et al. 2025; Li et al. 2023a).

Microbial community analysis revealed a reduction in *E. coli* and other conditionally pathogenic bacteria in two-stage fermentation, alongside increased abundances of *Lactobacillus*, *Bacillus*, and *Weissella.* These shifts are consistent with prior reports (Li et al. 2023b). The antimicrobial activity of *Bacillus* (Caulier et al. 2019) and lactic acid bacteria likely contributed to pathogen suppression (Caulier et al. 2019). Spearman’s correlation analysis further indicated that *Laciplantibacillus* abundance positively correlated with TAN, ASP, and enzyme activity, but negatively with TVBN, PUT, and SPE, consistent with previous studies (Dong et al. 2023; Luo et al. 2020; Zhao et al. 2022). *Bacillus* abundance positively correlated with TAN and amylase activity and negatively with MDA, aligning with findings by previous study (Peng et al. 2021). Conversely, *Acinetobacter* dominant in the N-group showed positive correlations with BAs levels and negative correlations with nutritional and enzymatic indices. Effective fermentation markedly suppressed *Acinetobacter* growth (Visca et al. 2011). These experimental data suggest that fermentation without probiotic supplementation results in the production of significant quantities of pathogenic bacteria and BAs, which accelerates feed spoilage. In contrast, fermentation supplemented with effective probiotics ensures successful fermentation.

Overall, two-stage fermentation with *B. subtilis* 3–16 and *L. plantarum* E5 effectively enhanced nutrient composition, enzymatic activity, antioxidant potential, and microbial quality of pig feed while reducing ANFs and harmful microbial metabolites.

## Conclusion

In conclusion, *B. subtilis* 3–16 exhibits great biological enzyme activity and effectively degrades antigenic factors. *L. plantarum* E5 produces strong acids and can effectively inhibit the growth of pathogenic bacteria. Our results showed that using a two-stage fermentation mode with 3–16 and E5 (first aerobic, then anaerobic) could significantly improve the nutrient content of complete pig feed. After fermentation, the CP, ASP, and TAN of the pig feed were significantly enhanced, and the antigenic proteins were almost completely degraded. Antioxidant and enzyme activities improved after fermentation. The number of pathogenic bacteria decreased significantly, while the number of beneficial bacteria increased significantly during fermentation. This study provides a foundation for the practical production and application of two-stage co-fermented liquid feed.

## Supplementary Information

Below is the link to the electronic supplementary material.

ESM 1 (PDF 182 KB)

## Data Availability

No datasets were generated or analysed during the current study.
