# Supplementary material for: Low-temperature two-stage probiotic fermentation enhances nutrition and safety of pig liquid feed
Source: Appl Microbiol Biotechnol. 2025 Nov 24;110:25. doi: 10.1007/s00253-025-13633-9 (PMC12812781; doi:10.1007/s00253-025-13633-9)
Supplement: Supplementary file 1 [file 253_2025_13633_MOESM1_ESM.pdf]

Supplemental Material

Applied Microbiology and Biotechnology

Low-Temperature Two-Stage Probiotic Fermentation Enhances Nutrition and Safety of Pig Liquid Feed

Aoran Zhang[1, a, b], Yuheng Cao[1, b], Yunfan Zheng[b], Limei Sun[b], Wang Yin[b], Jie Yu[c], Bing Yu[c], Lei Yan[b], Xue Yan[b], Yunxiang Liang[a], Xiaoqing Pu[b], Yunfang Song[b], Aibing Yu[*, b]

[a] State Key Laboratory of Agricultural Microbiology, College of Life Science and Technology, Huazhong Agricultural University, Wuhan, Hubei, 430070, China

[b] New Hope Liuhe Co., Ltd, Key Laboratory of Feed and Livestock and Poultry Products Quality & Safety Control, Ministry of Agriculture and Rural Affairs, Chengdu, Sichuan, 610023, China

[c] Animal Nutrition Institute, Sichuan Agricultural University, Chengdu, Sichuan, 611130, China

The e-mail address, telephone and fax numbers of the corresponding author:

[*]Corresponding author: Aibing Yu, Email: yuaibing@newhope.cn

Legends of table

Table S1. Antagonistic relationship of *Bacillus* strains and *Lactobacillus* strains

Table S1. Antagonistic relationship of *Bacillus* strains and *Lactobacillus* strains

**Groups**		**Result**	**Groups**		**Result**
**MB360**	E5	-	**F5001**	E5	+
	RE16	-		RE16	-
	RE4	-		RE4	+
	RB16	-		RB16	-
**Y28**	E5	+	**DP4-7**	E5	-
	RE16	+		RE16	-
	RE4	+		RE4	-
	RB16	-		RB16	-
**3-16**	E5	-	**P2**	E5	-
	RE16	-		RE16	-
	RE4	-		RE4	-
	RB16	-		RB16	-
**3-24**	E5	+			
	RE16	+			
	RE4	+			
	RB16	-			

Note: “+” indicates significant antagonism, “-” indicates not antagonistic
